# Characterization of the Pre-lens Tear Film in Hydrogel and Silicone Hydrogel Contact Lens Wear

**DOI:** 10.18502/jovr.v20.16812

**Published:** 2025-09-09

**Authors:** Andrea Novo-Diez, Laura Valencia-Nieto, Sara Pérez-Charro, Alberto López-de la Rosa, Alberto López-Miguel, María J. González-García

**Affiliations:** ^1^Institute of Applied Ophthalmobiology (IOBA), Universidad de Valladolid, Valladolid, Spain; ^2^Biomedical Research Networking Center in Bioengineering, Biomaterials and Nanomedicine (CIBER-BBN), Universidad de Valladolid, Valladolid, Spain

**Keywords:** Hydrogel Contact Lens, Lipid Layer Thickness, Pre-Lens Tear Film, Tear Break-up Time, Tear Evaporation Rate

## Abstract

**Purpose:**

Different contact lens (CL) materials have been associated with different behaviors of pre-lens tear film components in the short term. The purpose of this randomized crossover and double-masked study was to compare the effect of wearing hydrogel and silicone hydrogel CLs on pre-lens tear film status.

**Methods:**

Soft CL wearers were recruited and randomly fitted with a hydrogel (omafilcon A) and a silicone hydrogel (stenfilcon A) CL. Tear evaporation rate, non-invasive tear break-up time (NIBUT), tear film lipid layer thickness, and partial blink rate were measured without CLs and 30 minutes after the insertion of CLs. The outcomes were analyzed using repeated measures analysis of variance or the Friedman test.

**Results:**

Twenty-four CL wearers (6 men and 18 women) aged 23.3 ± 3.9 years were included. Tear evaporation rate was higher with the hydrogel CL (98.6 ± 59.4 g/m^2^/h; *P *= 0.043) and the silicone hydrogel CL (99.7 ± 60.6 g/m^2^/h; *P *= 0.037) compared to no CL wear (69.9 ± 41.3 g/m^2^/h). NIBUT was lower (*P *= 0.019) with the silicone hydrogel CL (12.7 ± 6.2 s) than with no CL wear (18.5 ± 9.8 s). Lipid layer thickness was lower with the hydrogel CL (64.9 ± 15.5) than with the silicone hydrogel CL (75.8 ± 14.0; *P* < 0.001) and no CL wear (75.9 ± 14.2; *P *= 0.001). No statistically significant differences were found in the partial blink rate.

**Conclusion:**

This study demonstrated that both hydrogel and silicone hydrogel CLs disrupt the tear film by increasing tear evaporation and causing tear film instability. However, CL materials affect the pre-lens tear film status differently. Further studies with longer wearing times are required.

##  INTRODUCTION

Contact lens (CL) wear divides the tear film into pre-lens and post-lens layers, causing a decrease in pre-lens thickness ranging from 2 to 7 µm.^[1,2]^ The insertion of a CL also induces changes in the composition and concentration of some tear film components,^[3,4,5]^ altering clinical parameters such as tear stability, lipid layer thickness, and tear evaporation rate.^[3]^ These biophysical changes have been previously related to CL discomfort symptoms.^[3]^

Tear film stability in CL wearers is commonly evaluated using non-invasive tear break-up time (NIBUT) measurement, which can predict CL discomfort.^[6,7,8]^ Previous studies have found a NIBUT decrease ranging from 5 to 7 seconds in symptomatic CL wearers compared to asymptomatic ones.^[7,9]^ It has also been reported that shorter NIBUT values are related to CL discomfort symptoms in both hydrogel and silicone hydrogel CL wearers.^[3,9]^ This reduction in NIBUT may be due to the reduced stability of the pre-lens tear film.^[10]^ Additionally, the lipid layer thickness is reduced in CL wearers,^[11,12]^ which in turn can decrease tear film stability, leading to an increased rate of tear evaporation.^[3]^ Specifically, when a CL is worn, the tear evaporation rate can increase by 1.2 to 2.6 times compared to when no CL is used.^[13]^ Therefore, CL wear can affect several aspects of the tear film that could lead to CL discomfort.

In addition to assessing the tear film during CL fitting, it is important to consider blinking dynamics, as its primary function is to evenly distribute the tear film across the ocular surface, creating a smooth and uniform surface for clear vision.^[14]^ A blink is considered partial when < 67% of the cornea is covered.^[3]^ Partial blinks are associated with CL discomfort and ocular dryness symptoms,^[3]^ possibly due to destabilizing the tear film and increasing the tear evaporation rate. Some studies have found no differences in the partial blink rate between hydrogel CL wearers and non-wearers.^[3]^ However, another study suggested that partial blinking is higher in CL wearers.^[15]^ Therefore, further research is needed on the relationship between different CL types and blink rate.^[16]^

Finally, the interaction between the CL and the pre-lens tear film also depends on the CL material. Silicone hydrogel CLs are more permeable to oxygen than hydrogel CLs. However, these materials might be more hydrophobic, which directly affects tear film properties.^[17]^ There are reports on differences in tear film stability between hydrogel and silicone hydrogel CLs.^[9,18,19]^ Nevertheless, limited data exist about their effect on the tear evaporation rate and lipid layer thickness during CL wear. Therefore, the present study aimed to compare the short-term effects of wearing hydrogel and silicone hydrogel CLs on pre-lens tear film status, as well as to evaluate tear film parameters in the absence of CL wear.

**Table 1:** Technical specifications of the contact lenses (CLs) used in the study

	**Hydrogel CL**	**Silicone hydrogel CL**
Laboratory	CooperVision	CooperVision
Replacement	Daily disposable	Daily disposable
Design	Single vision	Single vision
Material	Omafilcon A	Stenfilcon A
Technology	PC Technology	Aquaform
Diameter (mm)	14.2	14.2
Base curve (mm)	8.7	8.4
Water content (%)	60	54
Data obtained from https://coopervision.es		

##  METHODS

This research was approved by an independent ethics committee (the East Valladolid Health Area Ethics Committee, Valladolid, Spain, code: PI 21-2491) and conformed to the principles and applicable guidelines for the protection of human participants in biomedical research.

This prospective, randomized, crossover, double-masked study was performed in compliance with the tenets of the Declaration of Helsinki. Written informed consent was obtained from participants prior to enrollment.

### Subjects and Study Design

We recruited spherical single vision CL wearers over 18 years of age with a refraction between –0.25 and –12.00 diopters (D), since this was the available negative power range for the CLs used. The exclusion criteria were any alteration or ocular anomaly contraindicating the use of CLs, including history of ocular surface disease or meibomian gland dysfunction, history of any ocular surgery, any change in systemic medication affecting the tear film within the last 3 months, pregnancy, and breastfeeding. This information was collected in the anamnesis prior to the clinical tests.

Two different hydrogel daily disposable CLs were used in the study: a hydrogel CL (omafilcon A; Proclear 1 Day, CooperVision Laboratories, Pleasanton, CA, USA) and a silicone hydrogel CL (stenfilcon A; MyDay, CooperVision Laboratories, Pleasanton, CA, USA). The technical parameters of both CLs are detailed in Table 1.

Volunteers attended the study visit without having used any CL for at least 24 hours before. First, the inclusion and exclusion criteria were checked, and the clinical evaluation was performed without CL wear. Second, participants were randomly fitted with one of the CLs (hydrogel or silicone hydrogel CL) in a double-masked fashion. After 30 minutes, the clinical evaluation was performed with the first CL in place. Then, the other study CL was fitted, and the same clinical evaluation was performed after 30 minutes of CL wear.

### Clinical Evaluation

Using the Contact Lens Dry Eye Questionnaire (CLDEQ)-8, we evaluated the discomfort symptoms participants experienced with their habitual CLs. The cut-off value of this scale to detect symptomatic CL wearers is a score ≥ 12.^[20]^

The tear evaporation rate was evaluated using a closed-chamber evaporimeter, the Eye-VapoMeter (Delfin Technologies Ltd, Kuopio, Finland). Participants were first instructed to blink normally and maintain their primary gaze position while three consecutive measurements were taken. Then, they were asked to keep their eyes closed while another three consecutive measurements were performed. The mean for both scenarios was calculated. The tear evaporation rate was determined as the difference between both means, thereby eliminating the effect of eyelid skin and surrounding tissue evaporation to avoid bias in measuring this rate.

Tear film stability was evaluated by measuring the NIBUT using the EasyTear VIEW+ (EasyTear Ltd., Rovereto, Trento, Italy). Participants were asked to blink three times before the measurement was acquired. Three measurements were performed, and the mean value was recorded.

Lipid layer thickness and partial blink rate were evaluated with the LipiView II interferometer (Johnson & Johnson Vision, Santa Ana, CA, USA). Participants were asked to blink normally while a 20-second video was captured. The mean value of the lipid layer thickness and the percentage of partial blinks were recorded. All clinical measurements were performed first without CLs and then with each of the two CLs.

### Statistical Analysis

The R statistical package (version 4.1.2) was used to perform the statistical analysis. A minimum sample size of 19 participants was estimated to detect an effect size of 0.83 in tear evaporation rate using a paired Student's *T*-test, with a statistical power of 80% and a significance level of 0.05/3 (adjusted for multiple comparisons). Using the data reported by Thai et al,^[21]^ we calculated the effect size by considering the change in tear evaporation rate from no lens wear to Omafilcon A wear (8.99 ± 10.89 g/m^2^/h). Differences between the three situations (data obtained without CLs, with hydrogel CLs, and with silicone hydrogel CLs) were analyzed using a repeated measures analysis of variance (ANOVA). Both eyes were evaluated, but only one eye was randomly selected for statistical analysis. The assumptions of normality and sphericity were checked using the Shapiro-Wilk test and the Mauchly test, respectively. Normal variables were the tear evaporation rate and lipid layer thickness. Parameters that did not meet the previous assumptions (NIBUT and blink rate) were analyzed using the Friedman test. Post-hoc pairwise comparisons were assessed using the paired Student's *T*-test. Alternatively, the non-parametric Wilcoxon test was used when normality assumptions were not met, with the Hommel correction applied for multiple comparisons.^[22]^ *P*-values < 0.05 were considered statistically significant. Data were presented as mean ± standard deviation.

##  RESULTS

### Subjects

Twenty-four hydrogel CL wearers (6 men and 18 women) with a mean age of 23.3 ± 3.9 years (range, 20 to 37 years) were included in the study. The average CL use before the study was 8.5 ± 5.3 years (range, 0.9 to 24 years) while the average CL wearing time was 5.4 ± 1.9 days per week (range, 1 to 7 days) and 8.5 ± 3.1 hours per day (range, 2 to 15 hours). The mean CL power was –3.75 ± 2.25 D (range –1.00 to –10.50 D), and the average CLDEQ-8 score was 6.8 ± 4.6 (range, 0 to 15). Five participants were symptomatic CL wearers according to the CLDEQ-8.

### Clinical Evaluation

**Figure 1:**
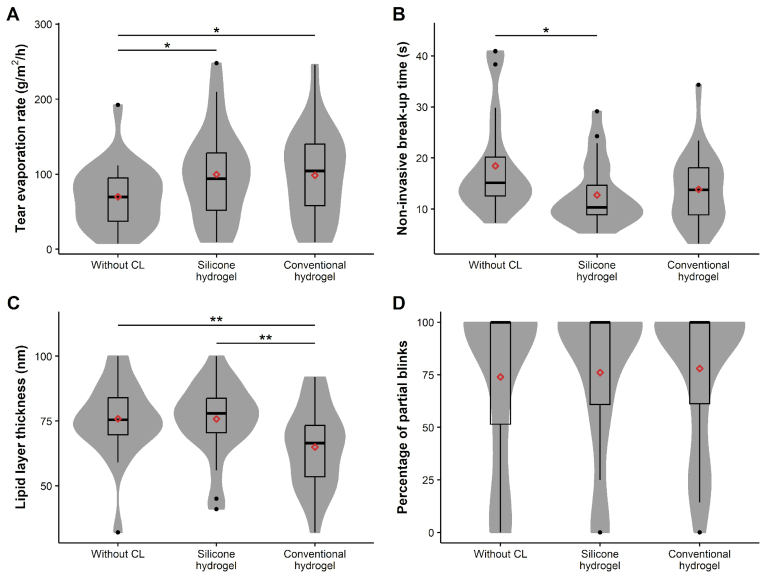
Measurements obtained during hydrogel and silicone hydrogel contact lens (CL) wear and in the absence of CLs. The shadows of the violin graphs represent the data density, and the red diamonds indicate the mean values (*P < 0.05, **P ≤ 0.001).

There were significant differences in tear evaporation rate measurements, as determined by ANOVA (*P *= 0.012). The multiple comparisons [Figure 1A] revealed a statistically significant increase in tear evaporation rate with both the hydrogel CL (98.6 ± 59.4 g/m^2^/h; *P *= 0.043) and the silicone hydrogel CL (99.7 ± 60.6 g/m^2^/h; *P *= 0.037), compared to the no CL condition (69.9 ± 41.3 g/m^2^/h). However, no differences were found between the two CL materials (*P *= 0.900).

According to the Friedman test, statistically significant differences in NIBUT were observed among the three scenarios (*P *= 0.018). A statistically significant difference (*P *= 0.019) revealed a lower NIBUT with the silicone hydrogel CL (12.7 ± 6.2 s) compared to no CL wear (18.5 ± 9.8 s) [Figure 1B]. No significant differences were found between the hydrogel CL (13.8 ± 6.9 s) and the no CL condition (*P *= 0.125), nor between the two CL materials (*P *= 0.384).

Significant differences in lipid layer thickness were found among the three scenarios (ANOVA *P* < 0.001). Lipid layer thickness was lower while wearing the hydrogel CL compared to the silicone hydrogel CL (64.9 ± 15.5 vs. 75.8 ± 14.0; *P* < 0.001) and the no CL condition (75.9 ± 14.2; *P *= 0.001). No differences were found between not wearing CLs and wearing the silicone hydrogel CL (*P *= 0.976) [Figure 1C].

Finally, there were no statistically significant differences in the partial blink rate observed among the three scenarios (Friedman test *P *= 0.477) [Figure 1D].

##  DISCUSSION

The insertion of a CL onto the ocular surface splits the tear film, altering its optimal structure and increasing its evaporation.^[1,2]^ Given that hydrogel and silicone hydrogel CLs are the most commonly fitted CLs,^[23,24,25]^ this study aimed to evaluate the pre-lens tear film changes in the ocular surface induced by CL wear, comparing a hydrogel CL (omafilcon A) and a silicone hydrogel CL (stenfilcon A).

The results of the present study confirmed that wearing a CL increases tear film evaporation; however, the tear evaporation rate did not differ between the two CL materials tested in the present study. The results align with those reported by Thai et al,^[21]^ who compared five CL materials (including omafilcon A) and found no significant differences among them. However, the results reported without CLs were lower in the study by Thai et al^[21]^ than in the present study (39.05 ± 19.03 g/m^2^/h vs. 69.9 ± 41.3 g/m^2^/h, respectively). The differences observed might be due to the use of different instruments to measure the tear evaporation rate. Thai et al^[21]^ used the Servo-Med EP-3 Evaporimeter, whereas we employed the Eye-VapoMeter in the present study. Another study using the Eye-VapoMeter also reported tear evaporation rate values in healthy individuals (range, 56.06 ± 25.61 to 73.31 ± 35.02 g/m^2^/h) that were similar to those observed in the present study.^[26]^

In the present study, a statistically significant decrease in NIBUT was observed with the silicone hydrogel CLs compared to no CL wear. Although no statistically significant differences were found between the hydrogel CLs and no CL wear, the differences may be considered clinically relevant, as NIBUT values were similar between the two CL materials. These findings align with other authors who reported decreased NIBUT values during the use of omafilcon A and stenfilcon A CLs compared to no CL wear.^[27,28]^

Lipid layer thickness was the only parameter that showed statistically significant differences between the two evaluated CLs. The lower value observed with the hydrogel CL compared to the silicone hydrogel CL and no CL wear was an unexpected finding. This discrepancy may be attributed to differences in the material composition (presence or absence of silicone in the polymer) or the hydration technology (Aquaform technology or PC Technology), both of which vary between the CLs used in this study. Designed to maintain hydration and wettability, these technologies can function differently, resulting in varied behaviors on the CL surface. As new technologies continue to emerge, independent evaluation of each material's performance is crucial. The results regarding NIBUT values suggest lower tear film stability during the use of silicone hydrogel CL, which could be due to a thinner tear film lipid layer. However, the difference in lipid layer thickness between no CL wear and silicone hydrogel CL wear was not statistically significant. This can be explained by the fact that the average lipid layer thickness values obtained in the present study were higher than those reported in other studies.^[29,30]^ This can reflect a characteristic of our study sample, consisting of young and asymptomatic participants with a large range of CL wear frequency. Further studies are needed to understand this contradictory result. The interaction between the tear film and the silicone hydrogel CL seems similar to that of the conventional hydrogel CL used in this study, so the final decision on whether to fit one CL or the other could be based on other properties, such as oxygen permeability.

Regarding the partial blink rate, no differences were found with the use of CLs or between the CL materials evaluated in the present study. The same results were reported in previous studies that compared partial blinks with and without CLs.^[15,31]^ These findings suggest that the hydrogel CL wear itself does not affect the area covered by the eyelids during blinking. Instead, the partial blink rate might be more influenced by the visual tasks performed during CL wear.^[32]^

The novelty of the present study, which makes it a well-controlled clinical experiment, lies in being the first to assess the tear evaporation rate to compare the tear film dynamics between a conventional and a silicone hydrogel CL in the same individuals in a randomized crossover and double-masked approach. However, this study has some limitations. First, clinical evaluation was performed after 30 minutes of CL wear to ensure lens stability. Hence, it may not be warranted to generalize the CL behavior observed in this study for a whole day of CL wear. It would be interesting to evaluate the tear film parameters and CL discomfort symptoms after longer hours of CL wear, as it is well known that CL discomfort increases throughout the day.^[33,34]^ Second, tear film measurements were performed during the same visit to facilitate the volunteer's recruitment. Therefore, although measurements were performed after 30 minutes of CL wear for both materials without a long washout time, tear film measurements with the second CL could be minimally affected after using the first one. However, both CLs were fitted following a random order. Thus, this methodology helped to avoid biased results. Finally, only two specific CLs—one hydrogel (omafilcon A) and one silicone hydrogel (stenfilcon A)—were evaluated. Therefore, while this study provides interesting insights for CL practitioners, it seems promising to conduct further studies that include different CL materials under different environmental conditions^[18,35]^ in order to objectively evaluate CL behavior based on tear evaporation rates and lipid layer thickness. Despite the above limitations, the present study indicates that it is necessary to consider all the properties of CLs when prescribing them. Silicone hydrogel CLs are excellent in terms of oxygen permeability, but some patients experience problems related to wettability, comfort, or vision. Thus, it might be recommended to routinely evaluate the tear evaporation rate and lipid layer thickness in clinical practice.

In summary, the insertion of a CL affects tear film properties in terms of evaporation and stability, but not blinking. However, different CL materials and hydration technologies induce different behaviors in the pre-lens tear film components, at least during short-term CL wear. Further studies are needed to assess whether these changes in tear film parameters persist after several hours of CL wear.

##  Financial Support and Sponsorship

This work was partially supported by the Ministry of Universities and European Social Fund under Grant number FPU19/01109; and Junta de Castilla y León and European Social Fund under Grant number EDU/556/2019. Funders had no role in the study.

##  Conflicts of Interest

None.
